# DEVELOPING AND TESTING THE FEASIBILITY OF A TEXT MESSAGE DEMENTIA CAREGIVER SUPPORT INTERVENTION FOR LATINOS

**DOI:** 10.1093/geroni/igac059.324

**Published:** 2022-12-20

**Authors:** Jaime Perales Puchalt

**Affiliations:** University of Kansas Alzheimer’s Disease Research Center, Kansas City, Kansas, United States

## Abstract

Latinos experience several disparities in dementia including poor caregiver mental health and caregiving support access. Latinos’ ubiquitous cell phone ownership and use of text messaging may offset those disparities. During this session, we will describe the development and feasibility testing findings of CuidaTEXT. CuidaTEXT is a tailored Short Messaging Service-based text message intervention to support Latino dementia family caregivers. This intervention was developed using mixed-methods research. This research resulted in a bilingual six-month intervention with automatic daily messages for all participants that address dementia education, self-care, social support, end-of-life, care of the person with dementia, behavioral symptoms, and problem-solving strategies. Participants who need more in-depth support, can use two functions: 1) every time they send a keyword-driven message (e.g., STRESS), they will receive an automatic message to support them with that topic, 2) for any other message they send (live chat), a coach will respond to their specific needs.

